# Microwave-Assisted Synthesis of Carbon Dot – Iron Oxide Nanoparticles for Fluorescence Imaging and Therapy

**DOI:** 10.3389/fbioe.2021.711534

**Published:** 2021-07-06

**Authors:** Seokhwan Chung, Miqin Zhang

**Affiliations:** Department of Materials Science and Engineering, University of Washington, Seattle, WA, United States

**Keywords:** carbon dots, fluorescence imaging, iron oxide nanoparticles, microwave-assisted synthesis, chemotherapy, drug delivery

## Abstract

Fluorescence microscopy is commonly used to image specific parts of a biological system, and is applicable for early diagnosis of cancer. Current fluorescent probes, such as organic dyes and quantum dots, suffer from poor solubility and high toxicity, respectively, demonstrating a need for a colloidal stable and non-toxic fluorescent probe. Here we present an iron oxide and carbon dot (CD) based nanoparticle (CNPCP) that displays optical properties similar to those of conventional fluorescent probe and also exhibits good biocompatibility. Fluorescent CDs were synthesized from glucosamine onto chitosan – polyethylene glycol (PEG) graft copolymer using microwave irradiation. These NPs were monodispersed in aqueous environments and displayed excitation-dependent fluorescence; they demonstrated good size stability and fluorescence intensity in biological media. *In vitro* evaluation of CNP as fluorescent probes in cancer cell lines showed that these NPs caused little toxicity, and allowed fast and quantitative imaging. Model therapeutic doxorubicin (DOX) was conjugated onto the NPs (CNPCP-DOX) to demonstrate the multifunctionality of the NPs, and *in vitro* studies showed that CNPCP-DOX was able to kill cancer cells in a dose dependent manner. These results indicate the potential of using CNPCPs as fluorescent probes capable of delivering chemotherapeutics.

## Introduction

Fluorescence microscopy is a powerful tool used for selective imaging of tissues, cells, and organelles. Combined with an excitable, stable fluorophore, it can be used for early diagnosis of diseases such as cancer (Jie et al., 2014; Pak et al., 2015; Wolfbeis, 2015). An ideal fluorophore should demonstrate good biocompatibility so that it does not interfere with any cellular processes, and exhibit fluorescence intensity sufficiently higher than any autofluorescence within the biological system for a more sensitive imaging system. The current state of the art fluorophores are organic dyes and metallic or semiconductor-based quantum dots (QDs) (Wegner and Hildebrandt, 2015; Yu et al., 2015; McHugh et al., 2018). However, many organic dyes contain hydrophobic functional groups, and hence suffer from poor solubility in aqueous environments. This requires them to be conjugated onto the surface of a biocompatible, water soluble carrier particle; they are also known to have poor photostability and are easily photo-quenched, making long-term imaging difficult (Wang et al., 2010; Grimm et al., 2015). On the other hand, QDs typically consist of heavy metals or semiconductors, which typically lead to severe levels of both short and long term toxicity (Bottrill and Green, 2011; Tavares et al., 2011; Jensen, 2012; Das and Snee, 2016).

Carbon-based nanomaterials have emerged as an alternative to heavy metal semiconductor QDs. Carbon dots (CDs) exhibit tunable optical properties, while lacking the toxicity associated with heavy metals and semiconductors in biological systems (Yang et al., 2009; Luo et al., 2014; Schroeder et al., 2016; Winkless, 2016; Iannazzo et al., 2017). These CDs consist of an amorphous carbon matrix core that consists of mainly sp3 hybridized carbons, but also contains sp2 domains and a passivated surface. The delocalized electronic states in the sp2 domains and the nature of the passivation on the surface can affect the photoluminescence of CDs (Li and Dong, 2018). There has been a number of reported methods for synthesis of CDs having various optical properties (Pan et al., 2010; Fan et al., 2013; Ahirwar et al., 2017). Compared to single- and multi-layered carbon nanotubes (SW/MWCNT), CDs are easier to synthesize, and their photoluminescence can be easily tuned, which has fueled the efforts at developing CD-based optical imaging systems for biological applications (Hu et al., 1999; Lin and Zhang, 2012).

Recent studies have focused on synthesis of CDs from relatively simple carbon sources, such as glucose, through microwave-assisted hydrothermal reactions. These methods use an aqueous solution of carbon-containing precursors that is irradiated with microwave radiation, and various separation techniques, such as centrifugation and column chromatography, are used to purify the CDs that display the desired properties (Tang et al., 2012, 2014). The microwave irradiation allows quick and uniform heating of the solution, which improves the homogeneity in physical and chemical properties. Furthermore, the aqueous nature of the reaction leads to water-soluble CDs that do not require further surface passivation due to the presence of hydrophilic functional surface groups. CDs can also be synthesized through other methods such as electrolysis and hydro/solvothermal treatment. However, these reactions do not allow precise control over physical and optical properties, and require strong oxidizing reagents as part of the synthesis process. Compared to these methods, the microwave irradiation yields more uniform products, and is a safer and faster approach to synthesis of CDs. Though the chemical reaction is complex and not yet fully understood, many reports suggest that glucose-based CDs are an ideal alternative to fluorophores (Agbenyega, 2012).

Iron oxide nanoparticles (IONP) are a versatile platform for a variety of biomedical applications due to their favorable properties such as good biocompatibility, versatile surface chemistry, and superparamagnetism. A typical IONP system consists of an iron oxide core that is coated by molecules such as polymers (Stephen et al., 2014, 2016), proteins (Huy et al., 2015), and small macromolecules such as glucose (Sun et al., 2009). These coatings provide various chemical functional groups on the surface of the IONPs, which improves their solubility, while providing sites for conjugation of targeting ligands (Sun et al., 2008), therapeutic drugs (Hwu et al., 2009; Kievit et al., 2011), and nucleic acids (Jiang et al., 2013; Stephen et al., 2016). In addition to the biochemical properties, IONPs with magnetite (Fe_3_O_4_) cores have superparamagnetic properties and have been developed as contrast agents for magnetic resonance imaging (MRI) (Laurent et al., 2008; Lee and Hyeon, 2012). For these reasons, IONPs have been widely investigated in cancer research as multifunctional nanomaterials, with applications such as tumor imaging (Ji et al., 2019) and drug delivery (Mansouri et al., 2017).

Here, we report a method for microwave-assisted synthesis of fluorescent CDs onto chitosan-PEG copolymer, which is then used to stabilize IONPs through a co-precipitation synthesis method to produce a dual imaging contrast agent. While IONPs are widely used as MRI contrast agents, they do not have inherent fluorescent property, limiting their utility to a single imaging modality. Previous reports of IONPs for multimodal imaging probes have utilized organic dyes on surface of core-shell nanoparticles. Significant fluorescence quenching of the dyes was observed in such designs (Jang et al., 2014). In this design, modification of chitosan-PEG with CDs allows the IONPs to be used as multimodal imaging agents, and no fluorescence quenching of the CDs was observed. Chitosan is well-known to be biocompatible, and contains functional groups which would allow conjugation of therapeutic molecules or ligands onto the surface of CNPCP. Previously, iron oxide nanoparticle coated with biocompatible chitosan-PEG (NPCP) without the CDs was reported to be a T_2_ MRI contrast agent (Sun et al., 2008; Stephen et al., 2019). CDs of small size are rapidly cleared by the body; the IONP as their host would greatly increase the residence time of the CDs *in vivo*, allowing long-term fluorescence imaging. The short reaction times and the availability of the precursors in the synthesis method make the method easy to scale up mass production. In this method, an aqueous solution of chitosan-PEG and glucosamine was irradiated inside a conventional microwave oven in presence of ammonia as a catalyst. The resulting polymer consisted of CDs formed from glucosamine on the chitosan-PEG, and fluorescent IONPs were synthesized through addition of ammonia into the solution of the modified polymer and ferrous and ferric ions. The physicochemical properties were analyzed using transmission electron microscopy, dynamic light scattering (DLS), and Fourier transform infrared spectroscopy. The optical properties of the NPs were optimized by varying the reaction conditions in the microwave and were analyzed using fluorescence spectroscopy and UV-Vis absorbance spectroscopy. Finally, *in vitro* experiments were performed to evaluate the NPs as fluorescence probes for cellular imaging. Toxicity, stability in biological media, and nanoparticle uptake in human glioma cells were assessed, and confocal fluorescence microscopy was used to evaluate the capability of NPs as fluorescence imaging probes. Furthermore, to demonstrate the utility of the design, DOX was conjugated onto the surface of the NPs as a model therapeutic. The therapeutic effect of the resultant NP-DOX conjugate was evaluated through assessment of cell viability after incubation with various doses.

## Materials and Methods

### Materials

All reagents were obtained from Sigma Aldrich (St. Louis, MO, United States) unless noted otherwise. Chitosan (MW 3900) was obtained from Acmey Industrial (Shanghai, China). Cell culture reagents including Dulbecco’s Modified Eagle Media (DMEM) and antibiotic-antimycotic solution were purchased from Invitrogen (Carlsbad, CA, United States). Fetal bovine serum (FBS) was purchased from Atlanta Biologicals. DOX was purchased from LC Laboratories (Woburn, MA, United States).

### Preparation Fluorescent Iron Oxide Nanoparticles (CNPCP)

Chitosan-PEG coated iron oxide nanoparticles with CD incorporated (CNPCP) were synthesized via co-precipitation as previously reported (Veiseh et al., 2009). Briefly, purified chitosan (3.9 kDa) and aldehyde-activated methoxy PEG were reacted via reductive amination to produce a PEG-grafted chitosan polymer (CP). To synthesize the CD-CP complex, purified CP (150 mg) and glucosamine hydrochloride (25 mg) was dissolved in deionized water. Diluted ammonia was added at 12.5 mg/ml. The solution was then transferred to a capped glass vial, and placed inside a conventional microwave. The vial was irradiated at 300W for varied duration. The resulting fluorescent CD-CP was purified through size exclusion chromatography in S-200 resin (GE Healthcare, Piscataway, NJ, United States) equilibrated with deionized water.

To synthesize the CNPCP, polymer mixture consisting of varied ratios of CP and CD-CP (150 mg total), iron (II) chloride (9 mg), and iron (III) chloride (15 mg) were dissolved in degassed deionized water (2.18 ml). Ammonia solution (36%) was titrated into the solution while being sonicated and stirred vigorously for 25 min under a nitrogen atmosphere. The ammonia was evaporated from the solution by continuing the sonication and stirring for additional 20 min to continue the growth of the nanoparticles. The resulting CNPCP were purified using S-200 resin equilibrated with deionized water.

### Characterization of CNPCP

Aqueous solution of CNPCP was diluted into a 50 mM HEPES buffer solution (pH 7.4), and hydrodynamic size and zeta potential of CNPCP were measured using a Zetasizer system. Electron micrographs of the samples were taken using a FEI Tecnai G2 F20 transmission electron microscope (TEM) (FEI, Hillsboro, OR, United States) operating at a voltage of 200 kV. The samples for TEM imaging was prepared by depositing 10 μL of the CNPCP solution onto a carbon-coated Cu 300 mesh grid. Fourier Transform InfraRed (FTIR) spectra of the samples were obtained with a Nicolet 6700 FTIR Spectrometer (ThermoFisher, Waltham, MA, United States). To minimize the interference from the iron oxide core, the core was dissolved with hydrochloric acid, and separated from the coating using a 3 k MWCO spin filter. The samples were then lyophilized and mixed into a KBr pellet at 0.2 wt%. Optical characterization was performed using fluorescence spectroscopy and UV-Vis absorbance spectroscopy. The fluorescence of the CNPCP solutions at various excitation wavelengths were recorded using a Horiba FL3-21tau Fluorescence Spectrophotometer (Kyoto, Japan). UV-Vis absorbance spectra of the CNPCP solutions were acquired using a UV-vis Spectrometer (Agilent Technologies, Santa Clara, CA, United States).

### Assessment of Stability in Biological Media

To assess the stability of CNPCP in biological media, CNPCPs were dispersed in DMEM with 10% FBS and 1% Penicillin-streptomycin, and incubated in water bath at 37°C for 14 days, during which DLS and fluorescence measurements were made every 2 days.

### Cell Viability Evaluation Through Alamar Blue Assay

SF763 human glioblastoma cells were cultured in DMEM supplemented with 10% FBS and 1% Penicillin-streptomycin. The cells were incubated at 37°C with 5% CO_2_ atmosphere. The effect of CNPCPs on viability of SF763 cells was determined using the alamarBlue assay following the manufacturer’s protocol (Life Technologies, Carlsbad, CA, United States). Briefly, cells were plated and treated with CNPCP as described. After treatment, cells were washed with phosphate buffered saline (PBS) three times before adding 10% alamarBlue solution in DMEM medium to the well. Cells were incubated for 24 h, then the alamarBlue solution was transferred to a 96-well plate, and the fluorescent emission at an excitation wavelength of 560 nm and an emission wavelength of 590 nm was read with a microplate reader.

### Evaluation of Cellular Uptake of CNPCP

SF763 human glioblastoma cells were seeded at 30,000 cells per well in a 24 well plate. Various concentrations of CNPCP were added to the wells. After 4 h of incubation time, the cells were collected, washed with PBS, and fixed in 4% formaldehyde (Polysciences, Inc., Warrington, PA, United States) for 30 min. Cells were then washed three times with PBS, and the cellular uptake was analyzed using the fluorescence of the CNPCPs through flow cytometry.

### Evaluation of CNPCP as Fluorescent Probes Through Confocal Microscopy

SF763 cells (50,000) were plated on a 24 mm glass coverslip and allowed to attach for 24 h. CNPCP solution was added to the cells, which were then incubated for 4 h. Afterward, cells were washed with PBS and fixed in 4% formaldehyde for 30 min. Cells were then washed three times with PBS, and coverslips were mounted on microscope slides. Images were acquired on an LSM 510 Meta confocal fluorescence microscope (Carl Zeiss, Inc., Peabody, MA, United States) with the appropriate filters.

### Preparation of CNPCP-DOX

To conjugate DOX onto CNPCP, DOX (1 mg) and succinimidyl iodoacetate (SIA) (0.57 mg) was reacted in dimethyl sulfoxide (DMSO) for 2 h. To activate the amine groups on CNPCP, 2-iminothiolane (1.18 mg) was added to 1.5 mg/ml of CNPCP in 0.1 M sodium bicarbonate, 5 mM EDTA buffer solution (pH 8.0) and reacted for 2 h. Activated CNPCP and DOX were mixed and reacted overnight at room temperature. Unreacted reagents were purified through size exclusion column chromatography using S-200 resin. Drug loading capacity was calculated by measuring the difference in absorption of CNPCP-DOX and CNPCP at 494 nm using a UV-vis Spectrometer (Agilent Technologies, Santa Clara, CA, United States).

### Evaluation of Therapeutic Effects of CNPCP-DOX

SF763 cells were seeded at 10,000 cells per well in a 96 well plate. Various concentrations of CNPCP-DOX were added to the wells. After treatment for 24 and 48 h, cells were washed with phosphate buffered saline (PBS) three times before adding 10% AB solution in DMEM medium to the well. Cells were incubated for 4 h, then the AB solution was transferred to a 96-well plate, and the fluorescent emission at an excitation wavelength of 560 nm and an emission wavelength of 590 nm was read with a microplate reader.

## Results

### Physiochemical Characterization of CNPCP

**FIGURE 1:**
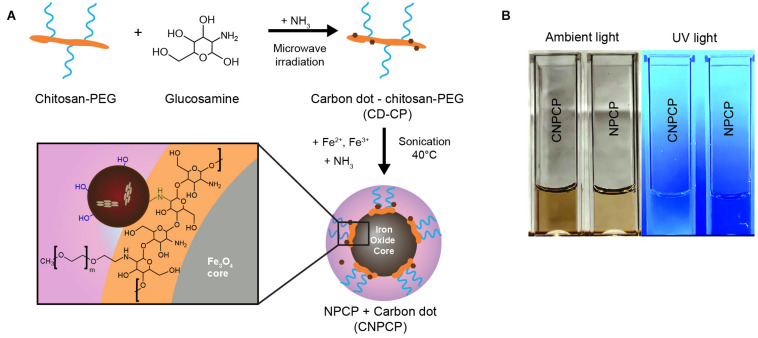
Overview of synthesis of CNPCP. **(A)** Schematic representation of synthesis of carbon dot-chitosan-PEG (CD-CP) and fluorescent iron oxide nanoparticle (CNPCP). **(B)** Images of aqueous solutions of CNPCP and NPCP.

Iron oxide nanoparticles coated with chitosan-PEG copolymer and carbon dots (CNPCP) were synthesized using a coprecipitation method. The chitosan-PEG coating provided the initial solubility in aqueous environments, and the steric stability imparted by the PEG kept the CNPCP well-dispersed without aggregation. The mechanism for the synthesis of CNPCP is illustrated in Figure 1A. The synthesized c-dots formed on the chitosan-PEG coating of the nanoparticle. The resulting solution was dark brown and highly fluorescent (Figure 1B).

**FIGURE 2:**
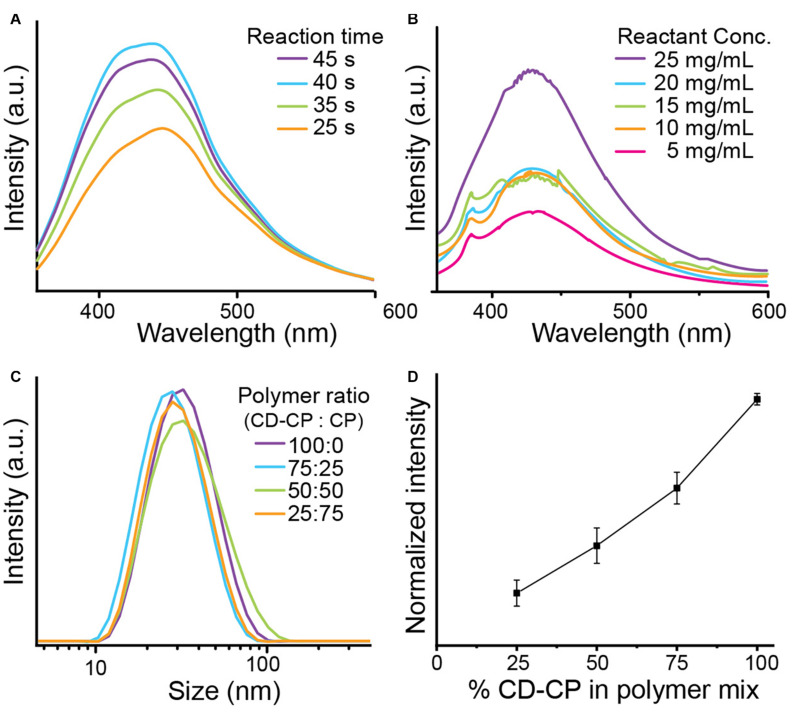
Optimization of CNPCP synthesis parameters. **(A)** CD-CP reacted for various lengths of time, and **(B)** CD-CP reacted with varied concentration of glucosamine. **(C)** Hydrodynamic size distribution of CNPCP made with varying ratio of CD-CP and CP. **(D)** Maximum fluorescence intensity with increasing CD-CP content in polymer mix.

The effect of varying parameters of the microwave-assisted reaction on the fluorescent properties of the resulting CNPCPs was investigated. The duration of irradiation in the microwave oven was first investigated. The concentration of reagents, as well as the incident power of the microwave irradiation was kept constant. The observed fluorescence spectra of CNPCP shows the fluorescence intensity increasing with longer times up to 40 s, after which the intensity slightly decreases (Figure 2A). Also observed with 45 s of microwave irradiation was a darker solution, followed by aggregation of the polymer after resting. At reaction times shorter than 25 s, no significant fluorescence was observed (data not shown), and at reaction times longer than 45 s, the solution was overheated and evaporated from the vial. This result shows that there is a critical temperature that the reaction must reach before formation of CDs on the polymer. However, prolonged irradiation leads to degradation of fluorescence and instability of the final product, likely due to uncontrolled carbonization of the reactants. Chitosan-PEG irradiated for 40 s was used for the remainder of the experiments.

The amount of glucosamine was also varied in the precursor solution, and all other parameters were kept constant. The resulting spectra from the samples shows that the fluorescence generally increased as the concentration of glucosamine was increased from 5 to 25 mg/ml (Figure 2B). A clear correlation was observed between the concentration of glucosamine and fluorescence intensity, with a sudden increase between 5 and 10 mg/ml, and between 20 and 25 mg/ml, indicating non-linear kinetics of CD growth with respect to the concentration of the precursor molecules. In contrast to the intensities, the window of the fluorescence emissions is shown to be largely unchanged with the variation of the reaction conditions.

To test whether using a mix of chitosan-PEG modified with CD (CD-CP) and unmodified chitosan-PEG (CP) would affect the stability of the synthesized CNPCPs, the hydrodynamic size of each batch of CNPCP made with varied ratio between CD-CP and CP was measured using DLS. As shown in Figure 2C, no significant differences were observed in the size distribution of the CNPCPs made with different ratio of CD-CP to CP. To investigate the effect of the polymer ratio on the optical properties of the resultant CNPCP, fluorescence intensities of CNPCP made with each polymer ratios were measured. While the trend shows that increasing the amount of CD-CP increases the fluorescence of the CNPCP, it can be seen that CNPCP synthesized with CD-CP only exhibits fluorescence most efficiently (Figure 2D). Using 75, 50, and 25% of CD-CP in the polymer mix yielded CNPCP that displayed 63.9, 40.6, and 21.3% of the fluorescence intensity exhibited by CNPCP made with 100% CD-CP.

**FIGURE 3:**
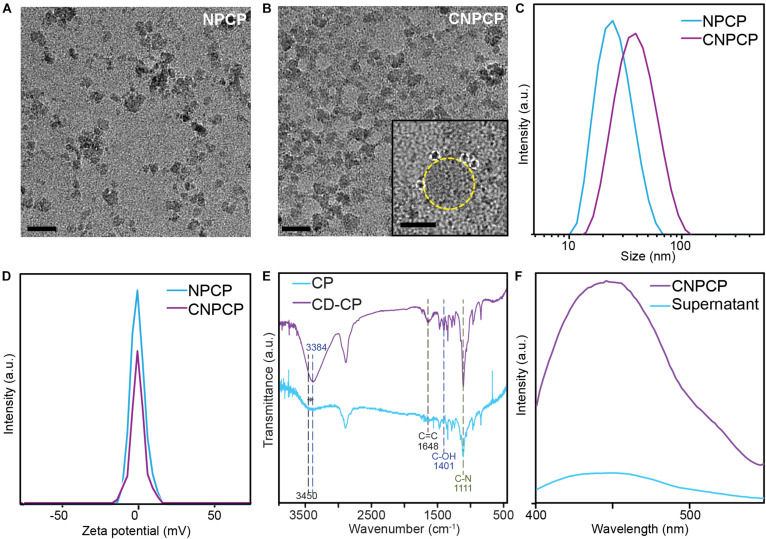
Physicochemical characterization of NPCP and CNPCPs. TEM images of **(A)** NPCP (synthesized without CD-CP), and **(B)** CNPCPs. CDs (circled with white dashed circle) can be found surrounding the iron oxide core of CNPCP (yellow) (inset). Scale bar = 20 nm, 5 nm in inset) **(C)** Hydrodynamic sizes and **(D)** zeta potentials of NPCP and CNPCP. **(E)** FTIR spectra of CD-CP and CP. **(F)** Fluorescence emission of CNPCP solution before (CNPCP) and after (supernatant) magnetic separation of CNPCP from the solution.

The parameters were optimized to synthesize stable and fluorescent CNPCPs, and transmission electron microscopy was used to observe the effect of modification of CP on the morphology and size of the iron oxide core. Figures 3A,B show the diameter of the iron oxide core to be 8.38 ± 2.32 nm; the CDs in the CNPCP are shown to be near the core of the nanoparticle with diameter 1.61 ± 0.31 nm (2b, inset). The hydrodynamic size of CNPCP was measured through DLS and was 38 nm, which was larger than the hydrodynamic size of 28 nm of NPCP prepared with the unmodified CP, but still monodisperse and smaller than 100 nm (Figure 3C). The monodispersity demonstrated by the CNPCPs indicates that while the CDs formed on the chitosan-PEG coating, the presence of the CD did not significantly affect the ability of the chitosan-PEG copolymer to form iron oxide nanoparticles in solution, and provide steric stability to the nanoparticle. The zeta potential measurements (Figure 3D) show that there was a decrease in the zeta potential from −1.01 to −1.24 mV after the reaction, indicating the presence of hydroxyl and carboxylic groups on the surface as a result of the decomposition of the glucosamine molecules. The presence of the functional groups on the surface of CNPCPs leads to high solubility in aqueous solutions, as well as provides sites for further conjugation with therapeutics and targeting ligands.

The chemical structure of the polymer coating and the CDs was investigated using FTIR spectroscopy, with the FTIR transmittance spectrum of CP as reference. Transmittance peaks at 1,648 cm^–1^ and 1,401 cm^–1^ correspond to C = C bonds and C–OH bonds, respectively, and are only present on the spectrum of the CNPCPs, indicating the formation of CDs. The structure of the chitosan-PEG copolymer does not contain any C = C bonds, and while chitosan contains hydroxyl groups, the peak at 1,401 cm^–1^ peak is observed in graphene-like structures, indicating the presence of a chemical structure that is different from the chitosan-PEG copolymer. The intensity of the 1,111 cm^–1^ peak that corresponds to C–N bonds is also greater in the CNPCP, indicating the formation of additional C–N bonds between the CDs and the chitosan backbone, as well as within the CDs. The shift of the peak from 3,450 to 3,384 cm^–1^ after the reaction also indicates the change in the amount of N–H and O–H bonds as the CDs are formed (Figure 3E).

The strong bond between the CD and the CP-coated nanoparticle was also investigated. A solution of CNPCP was placed above a strong magnet to separate the magnetic CNPCP from the solution, and the fluorescence of the resulting supernatant was measured to investigate the strength of the association between the CNPCPs and the CDs. As shown in Figure 3F, the supernatant demonstrated significantly lower fluorescence than that of the CNPCP solution. Since the CD are the primary source of fluorescence in CNPCP, this result indicates that much of the CDs were associated with the CNPCPs that were separated from the solution. While some CDs remained in the supernatant, as shown by the peak observed in the fluorescent spectrum of the supernatant, this shows that the CDs are strongly bound to the surface of the nanoparticles, as opposed to being loosely associated with the nanoparticles. This highlights the stability of the CNPCPs, which is important to the application of CNPCPs as imaging probes by ensuring strong, undiffuse signal.

### Optical Properties of CNPCP

**FIGURE 4:**
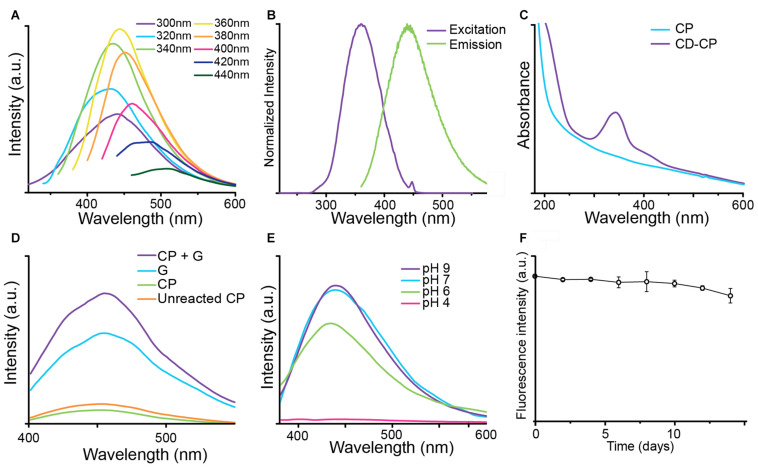
Optical properties of CNPCP. **(A)** Fluorescence emission spectra generated under different excitation wavelengths. The CNPCP display a strong fluorescence in the green-blue range of the spectrum. **(B)** Fluorescence excitation (purple) and emission (green) profiles of CNPCP. **(C)** UV-Vis absorbance spectra of CP and CD-CP. The CD-CP shows an absorbance peak around 340 nm, indicating the presence of CDs on the NPs. **(D)** Fluorescence emission from microwaved solutions of chitosan-PEG and glucosamine (CP + G), glucosamine (G), chitosan-PEG (CP), and unmodified chitosan-PEG (Unreacted CP). **(E)** Fluorescence spectra of CNPCP in aqueous solutions of various pH values. **(F)** Fluorescence intensity of CNPCP in DMEM incubated at 37°C over time.

The fluorescence of CNPCP was measured using various excitation wavelengths. Figure 4A shows the excitation-dependent fluorescence emission of CNPCPs. This wavelength-dependent tunable fluorescence is characteristic of many QD-based nanoparticle systems. The maximum fluorescence intensity was observed when an excitation wavelength of 360 nm was used (Figure 4A), which produced an emission spectrum with a peak around 450 nm (Figure 4B). As the iron oxide core contributes to the background absorbance in the UV-Vis spectrum, the absorbance spectra of the modified and unmodified polymers were recorded. The UV-Vis absorbance spectrum of the CD-CP contains a peak at 340 nm that is not present in that of CP, demonstrating a change in the optical properties with the formation of CDs in the CNPCPs (Figure 4C).

To investigate the origin of the fluorescence demonstrated by CNPCPs, CP, glucosamine, and a mixture of CP and glucosamine were reacted in the microwave. The resulting fluorescence spectra is shown in Figure 4D. The solution containing CP and glucosamine and glucosamine only displayed much greater fluorescence than that of the solution containing CP only. The combination of CP and glucosamine in the initial solution resulted in stronger fluorescence than the reaction of glucosamine by itself.

As the structure of CD contains labile protons, the pH of the aqueous environment and the subsequent protonation/deprotonation of these functional groups could lead to changes in the fluorescence of the CNPCP. To investigate this effect, CNPCP were placed in buffers of various pH. No significant photo-quenching was observed above pH 6; however, at low pH below 6, the fluorescence was quenched drastically (Figure 4E). It is important the CNPCPs be fluorescent between pH 6–8, as many physiologically relevant pHs fall within these values, and CNPCPs can be used as fluorescent probes in biological systems. The fluorescence of CNPCPs following incubation in cell culture media at 37°C was measured over time to assess the stability of the fluorescent CDs in biological conditions. Fluorescence intensity of the CNPCP solution in cell culture media was stable for up to 10 day, after which slight decrease in the fluorescence intensity was observed (Figure 4F).

### Cell Viability and Uptake of CNPCP

**FIGURE 5:**
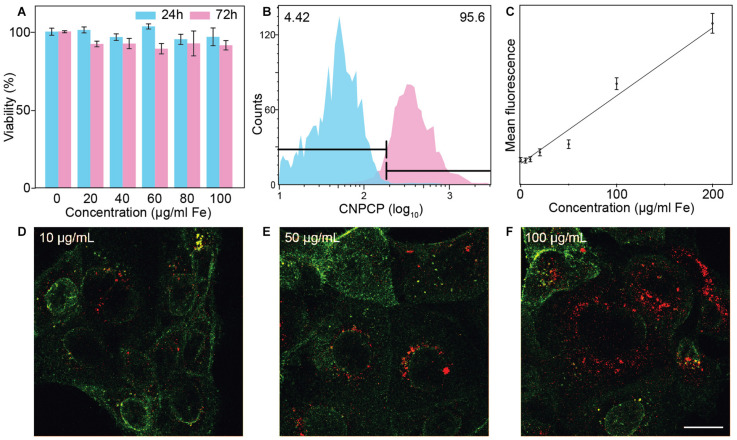
*In vitro* evaluation of CNPCP as fluorescence imaging probe. **(A)** Cell viability of SF763 cells evaluated via alamarBlue assay. **(B)** Representative histogram of distribution of cells treated (pink) with CNPCP and untreated (blue) cells. **(C)** Comparison of mean fluorescence and nanoparticle measured through flow cytometry. **(D–F)** Confocal fluorescence microscopic images of SF763 cells treated with NPCPs at 10, 50, and 100 μg/ml. WGA-AF647 membrane stain will show in green and fluorescence from CNPCP is shown in red. Scale bar = 20 μm.

AlamarBlue assay was used to assess the cell viability of SF763 cell line treated with CNPCPs. Though CNPCP demonstrated good water solubility and stability in cell culture media, it is important to assess potential toxicity of the nanoparticles for use in biological systems. The cells were treated at various concentrations of CNPCPs from 0 to 100 μg/ml. As can be seen in Figure 5A, the viability of the cells treated for 24 and 72 h both show minimal toxicity. As glucosamine, iron oxide, and chitosan are all known to be biocompatible, little to no cell killing was expected from these NPs. This design of chitosan-PEG coated IONP previously reported by our group was reported to be non-toxic, and many studies on CDs show that CDs cause little to no cytotoxicity *in vitro*. This further demonstrates that the CNPCPs are suitable as bioimaging probes, as they do not display high levels of cytotoxicity on their own.

The extent of cellular uptake of CNPCP was assessed through flow cytometry following incubation with varied concentrations of CNPCPs. The innate fluorescence of the CNPCPs allowed measurement of uptake without the need to conjugate additional molecular fluorescent dyes onto the nanoparticles. The SF763 cells exhibited high uptake of CNPCPs – incubation in 200 μg/ml of CNPCPs showed that 95.6% of the cells had internalized the nanoparticles (Figure 5B). At lower concentrations, as shown in Figure 5C, the mean value of the fluorescence observed from the cells correlated with the concentration of the CNPCPs. These results indicate that CNPCPs exhibit stability in biological media, do not elicit significant cytotoxicity, and are taken up by glioblastoma cells in a dose-dependent manner.

To demonstrate the capability of CNPCPs as fluorescence imaging probes, confocal images of SF763 cells treated with CNPCPs show that the CNPCPs have been internalized, with the greatest intensity observed around the nucleus (Figures 5D–F). Due to their small size and near-neutral surface charge, the CNPCPs were able to readily penetrate the cell membrane. No NPs were observed in the interior of the cell nuclei. NP systems commonly used to target the cell nucleus are either conjugated with targeting peptides or display highly positive surface charge to penetrate the nuclear membrane. Since there were no additional conjugation onto the CNPCPs, and zeta potential was measured to be near neutral, the NPs were expected to remain outside the nucleus. The intensity of the fluorescence from the CNPCP shows correlation with the nanoparticle uptake data, which indicates that the fluorescence of the CNPCPs were not affected by interaction with the intracellular components.

### CNPCP-Mediated *in vitro* Delivery of DOX

**FIGURE 6:**
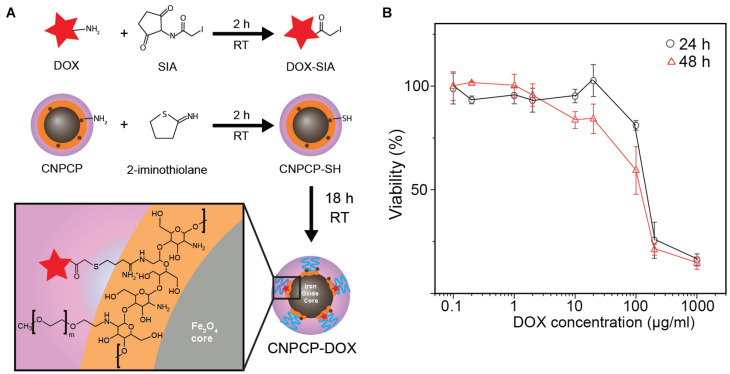
Chemotherapeutic delivery by CNPCP. **(A)** Schematic representation of conjugation of DOX onto CNPCP. **(B)** Cytotoxicity curve of CNPCP-DOX on SF763 after 24 and 48 h incubation.

In addition to their fluorescence, CNPCPs also contain functional groups which allow conjugation of therapeutic molecules. Doxorubicin (DOX) was conjugated onto the amine groups of the chitosan backbone (CNPCP-DOX) to demonstrate the utility of CNPCP in therapeutic applications (Figure 6A). The drug loading capacity was calculated to be 13.3% by measuring the absorption at 494 nm. The CNPCP-DOX was then added to SF763 cells which were incubated for 24 and 48 h. Viability of the cells was evaluated using alamarBlue assay, which showed that at concentrations above 100 μg/ml DOX, the proportion of healthy cells was much lower (Figure 6B). Since the CNPCPs were shown to be non-toxic, the therapeutic effect of CNPCP-DOX can be attributed to the conjugated DOX only. This result highlights the versatility and utility of the CNPCP design, which can be used in both imaging and therapeutic applications.

## Discussion

### Physiochemical Characterization of CNPCP

Stable and monodisperse fluorescent iron oxide nanoparticles coated with biocompatible chitosan-PEG polymer was synthesized using a coprecipitation method. To confer fluorescent properties to iron oxide nanoparticles which are not inherently fluorescent, the chitosan-PEG polymer (CP) was mixed with glucosamine and ammonia, and irradiated in a conventional microwave. Microwave irradiation onto various carbon-based precursor molecules have shown to produce CDs more rapidly than conventional means of synthesis (Choi et al., 2017; Xiao et al., 2017). The resulting complex of CD and CP (CD-CP) was used to stabilize the iron oxide nanoparticles during coprecipitation. Comparison of FTIR spectra (Figure 3E) and UV-Vis absorbance spectra (Figure 4C) between CD-CP and CP shows the presence of carbon double bonds and delocalized electrons in CD-CP, confirming the synthesis of CD through microwave irradiation. Though many studies have shown microwave-assisted synthesis of carbon-based materials, this unique design utilized CP as a substrate in the reaction, and used the resulting complex of CD-CP to stabilize iron oxide nanoparticles. The effect of the microwave-assisted modification of the CP on size and stability of the as-synthesized CNPCPs was also investigated.

Size and solubility are of utmost importance in the evaluation of nanoparticle for biological applications; NPs with sizes outside of a range of 10–100 nm will often be cleared out by the liver and the kidney, and NPs with poor solubility lead to aggregation, causing further complications (Shilo et al., 2015; Wang and Liu, 2018). As the CNPCPs have a hydrodynamic size within this range, they are expected to have minimal initial clearance in biological systems. The zeta potential can indicate the stability of the nanoparticle in the dispersion medium. Though a higher magnitude of zeta potential is associated with greater stability due to electrostatic repulsion between the nanoparticles, the CNPCPs show good stability in aqueous environments due to the PEG grafted onto the chitosan coating. The PEG provides steric stabilization of the CNPCPs, allowing them to have a small value of zeta potential and inhibiting the aggregation of the nanoparticles. Furthermore, nanoparticles with high zeta potential have been shown to cause acute cytotoxicity. As the zeta potential of the CNPCPs are close to neutral, they are not expected to cause significant cytotoxicity. These characteristics are conducive to applications of CNPCPs in biological aqueous environments.

### Optical Properties of CNPCP

Chitosan is known to exhibit fluorescence, which has been utilized in various applications such as biosensors and probes (Geng et al., 2015; Lee et al., 2017). To distinguish the inherent fluorescence of chitosan from that of the CDs in CNPCP, fluorescence intensities of solutions of CP, glucosamine, and a mixture of CP and glucosamine that were irradiated with microwave were compared to fluorescence intensity of unreacted CP (Figure 4D). While fluorescence intensities of the solutions containing glucosamine were increased, the solution that contained both CP and glucosamine displayed the greatest fluorescence intensity. This can be attributed to the heterogeneous nucleation of CDs on the CP, which requires less energy than the homogeneous nucleation in the solution containing glucosamine only. As a result, greater amount of CDs were formed in the solution containing CP and glucosamine. The CP copolymer serves not as a source of fluorescence, but as a substrate for the growth of CDs. Interestingly, the fluorescence of CP was observed to be diminished after the treatment in the microwave, likely due to quenching of the fluorescence at higher temperatures (Baker, 2005).

The CNPCPs display optical properties similar to those observed in CD systems, such as the excitation wavelength dependence of fluorescence emission spectra, and the characteristic absorbance peak at 340 nm. The fluorescence spectra displayed by the CNPCPs can be attributed to several factors including size effects, edge states, and functional groups (Sangam et al., 2018). While similarities in fluorescence emission of various carbon-based nanostructures have been observed, various explanations for the origin of fluorescence in CD structures have been proposed (Demchenko, 2019). One possible explanation of the origin of the fluorescence properties of the CDs on the CNPCPs could be that each CD acts as a quantum emitter, while the variation in size, surface properties, and composition between individual carbon nanostructure leads to a mixture of fluorescence emissions from the individual CDs (Demchenko and Dekaliuk, 2016). One of the important factors in determining the emission window of the fluorescence of carbon nanostructures is the composition of the precursors (Dey et al., 2014; Hasan et al., 2018). As demonstrated in the optimization of the reaction parameters, the chemical composition of the reagents remained unchanged in these experiments and no significant shift in the general fluorescence emission was observed.

To further investigate the fluorescence behavior of the CNPCPs, the fluorescence was measured in aqueous solutions of various pHs. The results showed that there were no significant photo-quenching observed above pH 6 (Figure 4E). According to previous reports, the PL mechanism of CDs are affected by proton concentration, as the edge functional groups can be protonated (Sangam et al., 2018). Deprotonation of these sites at low pH renders the photoluminescence of CDs inactive. Conversely, at high pH, the edge functional groups are protonated, and the PL of CDs is restored. It is important the CNPCPs be fluorescent between pH 6–8, as many physiologically relevant pHs fall within these values, and CNPCPs can be used as fluorescent probes in biological systems. While the fluorescence is quenched below pH 6, it was shown to be restored at higher pH, allowing the CNPCPs to be used as a pH sensitive on/off probe.

The UV-Vis absorbance spectrum of CNPCPs shows a peak at 340 nm, attributed to the π–π^∗^ transitions in the C = C bonds within the CDs (Figure 4C). Furthermore, a shoulder-peak is observed around 400 nm, indicating the n-π^∗^ of the C = O bonds. These absorbance peaks imply that the electronic transitions within the CDs provide delocalized π states in the basal plane, and the carbonyl or carboxylic groups produce the PL behavior observed in the CNPCPs.

### CNPCP as Multifunctional Imaging and Therapeutic Delivery System

The evaluation of cellular uptake of CNPCPs in SF763 highlighted the utility of the CNPCPs, as no fluorescent dye had to be conjugated onto the NP, and the inherent fluorescence of CNPCPs was used to analyze the population of treated cells. A linear trend is observed between the concentration of the CNPCP and the mean fluorescence of the treated cell, showing that the uptake and the resulting fluorescence of the cell is dose-dependent, and that the cellular uptake mechanism was not exhausted. Confocal fluorescence images of SF763 cells treated with CNPCP (Figures 5D–F) show that the regions with CNPCP can be distinctly identified in the images. The CNPCPs displayed consistent hydrodynamic size during incubation in 37°C, exhibited negligible toxicity, and was utilized as a fluorescent imaging probe.

The presence of functional groups on the chitosan presents opportunities to conjugate various therapeutics or targeting ligands onto the CNPCP to confer multifunctionality. To evaluate the effectiveness of CNPCP in therapeutic applications, doxorubicin (DOX) was chosen as the model therapeutic drug. DOX has been used previously to treat various cancer types; however, in the case of glioblastoma, it has seen limited use due to the high dosage required for systemic injection, and its inability to cross the blood-brain barrier. CNPCP-DOX induced toxicity at high concentrations and was able to kill tumor cells. In addition to delivery of DOX to glioma cells, the CNPCP was also able to aid in increasing the solubility of DOX in aqueous solution. In our previously reported study, iron oxide NP synthesized through co-precipitation and coated with chitosan-PEG was modified with chlorotoxin, a targeting peptide for glioma cells, was able to cross the blood-brain barrier (Veiseh et al., 2009). Because the CNPCP is synthesized from a similar design and materials, it is possible that CNPCP would also allow crossing of the blood-brain barrier for therapeutics that previously was limited by the this biological barrier.

In this study, chitosan-PEG co-polymer was modified with glucosamine via a microwave reaction to form carbon quantum dots in the polymer, and was used to synthesize fluorescent iron oxide nanoparticles (CNPCPs) that can be utilized as a fluorescent probe in biological systems. The NPs were small (under 100 nm) and showed good stability in cell culture media and minimal cytotoxicity *in vitro*. The NPs also showed great optical properties, as measured through fluorescence spectroscopy and UV-Vis absorbance spectroscopy. Cell viability results suggested that the CNPCPs were biocompatible and non-toxic, as no significant changes in cell viability was found both 24 and 72 h after the cells were treated with the CNPCPs. The CNPCPs were shown to be internalized within the cytoplasm in cells by fluorescence imaging, and the greatest intensity (i.e., the highest NP accumulation) was observed around the nucleus. These results show that the CNPCPs are capable of being used as a fluorescence imaging probe and could potentially be used for nuclear targeting with conjugation of targeting ligands. The versatility of CNPCPs was evaluated through conjugation of DOX as a model therapeutic. CNPCP-DOX was able to kill SF763 glioma cells at concentrations above 100 μg/ml. Through this study, we demonstrated a simple and yet effective nanoparticle synthesis approach using a conventional microwave to produce a fluorescence imaging probe. This synthesis approach holds a great potential at developing nanoparticle systems that could be used for not only biological imaging, but also therapeutic delivery and biosensing.

## Data Availability Statement

The raw data supporting the conclusions of this article will be made available by the authors, without undue reservation.

## Author Contributions

SC and MZ designed the project. SC prepared the materials, performed the measurements, and analyzed the data. Both authors have read and agreed to the published version of the manuscript.

## Conflict of Interest

The authors declare that the research was conducted in the absence of any commercial or financial relationships that could be construed as a potential conflict of interest.
